# Supplementary material for: Start codon disruption with CRISPR/Cas9 prevents murine Fuchs’ endothelial corneal dystrophy
Source: eLife. 2021 Jun 8;10:e55637. doi: 10.7554/eLife.55637 (PMC8216720; doi:10.7554/eLife.55637)
Supplement: Transparent reporting form [file elife-55637-transrepform.docx]

***eLife’s* transparent reporting form**

We encourage authors to provide detailed information *within their submission* to facilitate the interpretation and replication of experiments. Authors can upload supporting documentation to indicate the use of appropriate reporting guidelines for health-related research (see [EQUATOR Network](http://www.equator-network.org/%20)), life science research (see the [BioSharing Information Resource](https://biosharing.org/)), or the [ARRIVE guidelines](http://www.plosbiology.org/article/info:doi/10.1371/journal.pbio.1000412) for reporting work involving animal research. Where applicable, authors should refer to any relevant reporting standards documents in this form.

If you have any questions, please consult our Journal Policies and/or contact us: [editorial@elifesciences.org](mailto:editorial@elifesciences.org).

**Sample-size estimation**

- You should state whether an appropriate sample size was computed when the study was being designed
- You should state the statistical method of sample size computation and any required assumptions
- If no explicit power analysis was used, you should describe how you decided what sample (replicate) size (number) to use

Please outline where this information can be found within the submission (e.g., sections or figure legends), or explain why this information doesn’t apply to your submission:

For in vitro experiment, we did not use any power analysis.

For in vivo experiment, to minimize the animal number, we use at least four different mice in each group, which is the minimum size giving p<0.05 when we adopt non-parametric test.

**Replicates**

- You should report how often each experiment was performed
- You should include a definition of biological versus technical replication
- The data obtained should be provided and sufficient information should be provided to indicate the number of independent biological and/or technical replicates
- If you encountered any outliers, you should describe how these were handled
- Criteria for exclusion/inclusion of data should be clearly stated
- High-throughput sequence data should be uploaded before submission, with a private link for reviewers provided (these are available from both GEO and ArrayExpress)

Please outline where this information can be found within the submission (e.g., sections or figure legends), or explain why this information doesn’t apply to your submission:

The experiment was normally performed twice per week.

The information about replication can be found within the section of Materials and Methods, figure legends and the source data files.

High-throughput sequence data is uploaded to GEO as GSE146999.

Any reviewers can access the data with the token qvmbqoustdobjut.

**Statistical reporting**

- Statistical analysis methods should be described and justified
- Raw data should be presented in figures whenever informative to do so (typically when N per group is less than 10)
- For each experiment, you should identify the statistical tests used, exact values of N, definitions of center, methods of multiple test correction, and dispersion and precision measures (e.g., mean, median, SD, SEM, confidence intervals; and, for the major substantive results, a measure of effect size (e.g., Pearson's r, Cohen's d)
- Report exact p-values wherever possible alongside the summary statistics and 95% confidence intervals. These should be reported for all key questions and not only when the p-value is less than 0.05.

Please outline where this information can be found within the submission (e.g., sections or figure legends), or explain why this information doesn’t apply to your submission:

All statistical tests and p values are noted in figure legends and in the “statistics analysis” section of the Materials and Methods.

(For large datasets, or papers with a very large number of statistical tests, you may upload a single table file with tests, Ns, etc., with reference to sections in the manuscript.)

**Group allocation**

- Indicate how samples were allocated into experimental groups (in the case of clinical studies, please specify allocation to treatment method); if randomization was used, please also state if restricted randomization was applied
- Indicate if masking was used during group allocation, data collection and/or data analysis

Please outline where this information can be found within the submission (e.g., sections or figure legends), or explain why this information doesn’t apply to your submission:

This information does not apply to this submission.

**Additional data files (“source data”)**

- We encourage you to upload relevant additional data files, such as numerical data that are represented as a graph in a figure, or as a summary table
- Where provided, these should be in the most useful format, and they can be uploaded as “Source data” files linked to a main figure or table
- Include model definition files including the full list of parameters used
- Include code used for data analysis (e.g., R, MatLab)
- Avoid stating that data files are “available upon request”

Please indicate the figures or tables for which source data files have been provided:

In all Figures we show raw data with error bars (standard deviation).

We uploaded the following relevant additional data files.

Figure3-figure supplement1_source data.xlsx

Figure4-source data 1.xlsx

Figure4-source data 2.xlsx

Figure4-figure supplement3_source data.xlsx

Figure5-source data.xlsx

Figure6_source data.xlsx

Figure7_source data.xlsx

Figure8_source data.xlsx

Figure9_source data.xlsx

Figure10_source data.xlsx

Figure10-figure supplement1_source data.xlsx

Figure10-figure supplement2_source data.xlsx

Table1-source data.xlsx

Table2-source data.xlsx

High-throughput sequence is uploaded to GEO as GSE146999 (see above).
